# Long-term cost-effectiveness of quality of diabetes care; experiences from private and public diabetes centers in Iran

**DOI:** 10.1186/s13561-022-00377-9

**Published:** 2022-08-19

**Authors:** Rahill Sadat Shahtaheri, Yahya Bayazidi, Majid Davari, Abbas Kebriaeezadeh, Sepideh Yousefi, Alireza Mahdavi Hezaveh, Abolfazl Sadeghi, Ahmed Hayder Mohsin aL Lami, Hadi Abbasian

**Affiliations:** 1 Department of Pharmacoeconomics and Pharmaceutical administration, Faculty of Pharmacy, Tehran University of Medical Sciences, Tehran, Iran; 2 Faculty of pharmacy and pharmaceutical science, Islamic adad university, Tehran, Iran; 3 Department of Public Health, Tehran University of Medical Sciences, Tehran, Iran; 4 Faculty of Pharmacy, Tehran University of Medical Sciences, Tehran, Iran

**Keywords:** Type 2 diabetes mellitus, Localized UKPDS model, Private sector, Public sector, Cost-effectiveness analysis, Quality of care, Iran

## Abstract

**Background:**

The quality of health care has a significant impact on both patients and the health system in terms of long-term costs and health consequences. This study focuses on determining the long-term cost-effectiveness in quality of diabetes care in two different settings (private/public) using longitudinal patient-level data in Iran.

**Methods:**

By extracting patients intermediate biomedical markers in under-treatment type 2 diabetes patients(T2DP) in a longitudinal retrospective study and by applying the localized UKPDS diabetes model, lifetime health outcomes including life expectancy, quality-adjusted Life expectancy (QALE) and direct medical costs of managing disease and related complications from a healthcare system perspective was predicted. Costs and utility decrements had derived on under-treatment T2DP from 7 private and 8 Public diabetes centers. We applied two steps sampling mehods to recruit the needed sample size (cluster and random sampling). To cope with first and second-order uncertainty, we used Monte-Carlo simulation and bootstrapping techniques. Both cost and utility variables were discounted by 3% in the base model.

**Results:**

In a 20-year time horizon, according to over 5 years of quality of care data, outcomes-driven in the private sector will be more effective and more costly (5.17 vs. 4.95 QALE and 15,385 vs. 8092). The incremental cost-effectiveness ratio (ICER) was $33,148.02 per QALE gained, which was higher than the national threshold.

**Conclusion:**

Although quality of care in private diabetes centers resulted in a slight increase in the life expectancy in T2DM patients, it is associated with unfavorable costs, too. Private-sector in management of T2DM patients, compared with public (governmental) diabetic Centers, is unlikely to be cost-effective in Iran.

## Introduction

Worldwide, diabetes mellitus (DM) is a major public health problem. The prevalence of T2DM in Iran was 11.4% in the adult population in 2011, with a growth rate of 35% during 2005–2011 [1]. This notable increase in diabetes prevalence reflects that Iran has a high diabetes burden, especially when considering the impact of complications related to diabetes [2–4]. Diabetes complications have a negative impact on the quality of life (QoL), and is also a significant source of medical costs in people with diabetes [5].

The delivery and access to a high quality of diabetes care can decrease the risk of microvascular and macrovascular complications and mortality [6], Therefore, improving the Quality of diabetes care, including maintenance of blood glucose, blood pressure, LDL, and other bio-markers on the optimal level, is crucial [7–10]. Health care providers can play a critical role in improving the diabetes quality of care [11]. Improving service quality can increase customer trust and loyalty, profitability and cost reduction for an organization/health profession, and ultimately achieving a competitive advantage [12]. Several studies have identified significant gaps between private and public sectors in their costs and quality of care [13–16].

Iran’s healthcare system is a public-private partnership. According to statistics, about 62% of institutions belong to medical colleges, 16% are in the private sector, 8% are in social security and the rest are in other organizations [17, 18]. In Iran, the public health sector has access to valuable resources and facilities, including professional workforce, medical equipment and technology, data sources and governmental budget. In contrast, Iran’s private healthcare sector has high momentum and an excellent reputation among the population, who see the area as the first choice for cutting-edge medical services [19]. In the private sector, where medical tariffs are typically higher,the markup difference from the public sector tariffs is covered by a patient’s co-payment.

The national program for the prevention and control of T2DM was designed in 1996. There are three levels of diabetes care in the enhanced hierarchical model on the urban phase in the Iranian Program on Prevention and Control of Diabetes (NPPCD-2010) [20]. At the Primary level, called the “diabetes unit” diabetes care includes urban health centers and private clinics for screening and patient care. Diabetes centers are at secondary level and include public and private hospital and specialty polyclinics. The tertiary level provides specialized care and includes specialized and subspecialty hospitals [21]. Primary, secondary, and tertiary diabetes care services are provided by the public sector, but over the last two decades, the government’s focus on primary health care has made the public sector the leading provider of primary health care in the country. Nevertheless the private sector plays an important role in providing secondary and tertiary health care in urban areas [22]. Our study aimed to evaluate the costs and health outcomes T2DM patients referred to public and private diabetes centers in Iran, which may have lessons for other developing countries.

## Methods

### Study design

We performed a cost-effectiveness analysis of diabetes care in private and public diabetes centers using patient-level data. The intermediate bio-markers were used as surrogates for the final outcomes in the model: These biomarkers were included HbA1c, LDL-cholesterol, systolic blood pressure, smoking status, HDL cholesterol, peripheral vascular disease, atrial fibrillation, weight, albuminuria, heart rate, white blood cell count, hemoglobin, and eGFR. The biomarkers were extracted from the patient’s profiles. We used the localized UKPDS outcomes model version 2 to calculate the total disease burden over an extrapolated lifetime for populations with type 2 diabetes (UKPDS Outcomes Model Copyright© 2005–2020 Innovation Ltd. of Oxford University).

### Structure of the model

The UKPDS^1^ Outcomes Model is a computerized Microsimulation tool designed to estimate essential health economics outcomes including: Life Expectancy, Quality-adjusted Life Expectancy (QALE), and the cumulative costs of complications within type 2 diabetes mellitus patients. The UKPDS Model uses a wide variety of input data, including patient’s baseline characteristics, previous clinical events, and can take into account changes in risk factor levels over time.

This model is used for economic evaluations by estimating changes in outcomes when risk factors such as blood glucose level, blood pressure, lipid levels, and smoking status changed. The model  predicts the results for the next 20 years and estimates confidence intervals around outcomes of interest and to cope with the first and second-order uncertainty. Statistical techniques, including Monte-Carlo simulation and Bootstrapping are applied in the model. Instructions on how to use the model have already been poblished [23].

### Sampling method

The prediction was based on Clinical data that were extracted from paper records on 15 diabetes centers in five provinces in Iran.

Using analytical insight and the cluster sampling method, we selected five provinces and included 15 main diabetes care centers (public, semi-public (centers related to the social security insurance organization) and private) in our analysis. Two steps sampling were applied to recruit the required sample size. In the first step, cluster sampling method was applied to select provinces (clusters). Tehran and Isfahan provinces are two metropolises (23% of Iran’s total population lived in these two provinces in 2016). These provinces have better access to specialized health care services compared to others. Yazd had the highest prevalence of diabetes (16.3%) in Iran. The family physician program is running in Mazandaran, and kurdistan is one of the deprived provinces regarding access to health care. In the second step, 15 main diabetes care centers selected from the provinces. The subjects from each center were selected using a random sampling method based on the patient identification number. In centers with statistically small populations, the total population was included.

In our hypothetical cohort simulation, we supposed that under-treatment patients in the public/private sector would follow their specialized treatment from the same ward if their disease progressed and needed higher-level services/facilities.

### Inclusion and exclusion criteria

Diagnosis of T2DP, using antidiabetic medications, regular visits for glycemic control, regular laboratory tests, and had being under observation throughout the study schedule were inclusion criteria for the patients.

### Costs

We considerd direct medical costs from payer perspective. The main components of the costs were intervention costs, surgical procedures costs, drug acquisition costs, visits, para-clinical diagnostic tests, and annual costs associated with monitoring patients and management of diabetes-related complications (i.e. ischemic heart disease, myocardial infarction, heart failure, stroke, renal failure, retinopathy, amputation, and foot ulcer). Patients’ medical records were used to extract health care utilization data. in order to extract annual direct medical costs a micro-costing approach was carried out. The costs of private cares were calculated based on the private sector medical tariffs for hospital treatments and diagnostic services, medical inpatient care, laboratory, imaging, and paraclinical services. Costs in the following years were assumed to be equivalent to the average costs of a diabetic patient in the year after diagnosis of diabetes (Table 1). Both cost and utility variables were discounted by 3% in the base-case model. All costs were calculated based on the 2017 U.S. dollar value.

**Table 1:** Modelled management costs and utility decrements

Year	1					≥ 2	
Condition	public		private		Utility decrement	Annual cost (US$)	Utility decrement
	Annual cost (US$) Acute	Annual cost (US$) Not acute	Annual cost (US$) Acute	Annual cost (US$) Not acute			
Ischemic artery disease	616.87	1280.15	1603.862	3328.39	−0.010	338.81	0.000
Myocardial infarction	2018.98	2609.39	5249.348	6914.41	−0.148	527.14	−0.060
Heart failure	-	2577.25	-	6700.85	−0.071	1288.63	−0.185
Stroke	743.07	1650.99	1900	4292.5	−0.165	430.44	−0.165
Amputation	-	1363.57	100	3548	−0.200	368.53	−0.172
Blindness	-	434.91	-	1130.76	0.131	144.97	−0.103
End-stage renal disease	-	2886.87	-	7505.86	−0.330	1934.49	−0.330
Diabetic wound	-	907.31	-	1900	−0.200	193.04	−0.210

### Outcomes

QALE was the primary outcome measure in this analysis (QALE). QALE is the remaining number of Quality-Adjusted life-Years at a certain age. It has been calculated from age-specific mortality rates and average Health-related Quality of Life (HrQoL) [24, 25]. As a simple, preference-based measure of HrQoL, the Euro Qol Group has developed the EQ-5D-3L questionnaire (Health-related Quality of Life) [26, 27]. The Persian-translated EQ-5D-3L questionnaire used to measure HRQOL through face to face interviews. We used a disutility approach, which represents a decrease in quality of life in patients with T2DM due to their disease status and complications. The disutility scores of the selected complications for Iranian population are not yet available, so we used the UK value set for evaluation of EQ-5D-3L questionnaire.

### Sensitivity analysis

Sensitivity Analysis(SA) shows how a model’s outputs are affected by variation in inputs to reflect uncertainty around the output. We carried out one-way deterministic SA to increase understanding on which variables have the largest impact on Expected Value and to specify the relative importance of patient characteristics in driving aggregate outcomes of life expectancy. We investigated the impact of change in risk factors, such as HbA1c, on patients remaining life expectancy by ±1 SD of the mean and of doubling and halving the rates of binary variables such as macroalbuminuria.

## Results

### Population

The characteristics of 1978 patients with T2DM referred to private and public diabetes centers were shown in Table 2.

**Table 2:** Demographic characteristic of patients with type 2 diabetes (*N* = 1978)

Sector		Private	Public
Percentage of Female		56	51
Mean diabetic age (years)		15.84	14.34
Average age (years)		62.85	63.45
Body Mass Index (percentage)	< 18.5 kg/m^2^	2	1
	18.5–24.9 kg/m^2^	19	19
	25.0–29.9 kg/m^2^	43	40
	≥30 kg/m^2^	36	40
Age (percentage)	< 45	5	10
	45–65	65	55
	> 65	30	35

Females held 56% of the total, and patients’ mean diabetes age was 15.8 and 14.3 years for women and men, respectively. From the total, 43 and 36% of the patients were overweight and obese, respectively. The age group classification showed 65% of women and 55% of men were in the age group of 45–65 years.

### Predicted costs of management and treatment

Table 3 showed the costs associated with managing diabetes-related complications during the prediction period (20 Years).

**Table 3:** Average costs for T2DM patients (20-year prediction), *N* = 1978

Health Care sector	Treatment Costs (US$)	Managing complications (US$)	Total cost (US$)
Private	2557.55	12,827.78	15,385.33
Public	1861.76	6231.01	8092.76
Total	2209.65	9529.39	11,739.05

The average treatment cost was $2557 and $1861 per patient treated in the private and public sectors, correspondingly. The average private and public sectors cost of managing complications were $12,827.78 and $6231 per patient, respectively. (The total average cost of managing complications was $9529.39). The average total costs were $11,739. The private and public sectors average total costs were $15,385 and $8092.76 per patient, respectively.

### Predicted outcomes

Table 4 summarized the mean LE and QALE during the prediction period (20 Years).

**Table 4:** Average clinical outcomes for T2DM patients (20-year prediction), *N* = 1978

Health Care sector	Life expectancy	QALE
Private	6.99	5.17
Public	6.77	4.95
Total	6.88	5.06

The average life expectancy gains were 6.88 years. It was 6.99 and 6.77 in the private and public sectors, respectively. The average QALE was 5.17 and 4.95 in the private and public sectors, respectively.

#### Sensitivity analysis

**Fig. 1:**
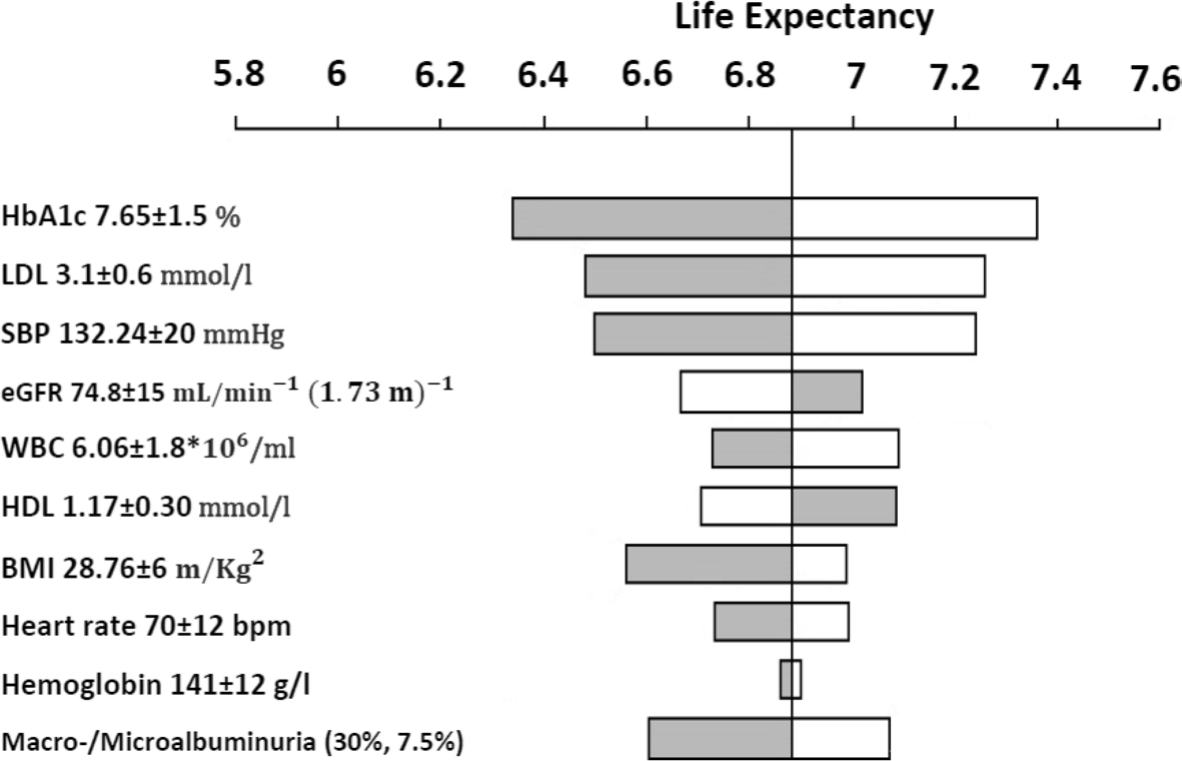
Result of the one-way sensitivity analysis

the results of the sensitivity analysis is shows in a tornado diagram (Fig. 1).

classical risk factors (HbA1c, SBP and lipid profile) has the largest impact on Expected Value. It as well shows the relative importance of other risk factors, in particular Body mass index, and micro/ macroalbuminuria on patients life expectancy. By contrast, eGFR, WBC, HDL, and haemoglobin had less impact on aggregate health outcome.

##### Base case analysis

The results of the cost-effectiveness of private versus public sector in T2DM patients from a payer perspective showed in Table 5. In a 20-year time horizon, the difference between QALE gains and direct medical cost in private and public diabetes centers is 0.22 years and 7292.56 $ in private and public diabetes centers, respectively.

**Table 5:** Base-case analysis per 1978 patients: Costs, Outcomes, and ICER of private sector compared with public sector diabetes care

Outcome	Private sector	Public sector	Difference
Life expectancy	6.99	6.77	0.22
QALE	5.17	4.95	0.22
Treatment costs (US$)	2557.55	1861.76	695.79
Costs of managing complications (US$)	12,827.78	6231.01	6596.77
Total costs (US$)	15,385.33	8092.76	7292.56
**ICER (US$)**			**33,148.02**

The incremental cost-effectiveness ratio (ICER) was $33,148.02 per QALE gained. In order to evaluate the estimated ICER for Iran, the result was compared with the recommende threshold of the World Health Organisation (WHO) for developing countriesa threshold. Iran's per capita GDP at the time of the study was $5,417.

## Discussion

The use of private care has always been an important issue for the treatment and management of diseases, especially the management of long-term diseases like diabetes. It has often been expected that private cares are more effective if they are more expensive. However, the question is how much more effective and how much more expensive they are. The focus of this study was on predict the long-term costs and outcomes of private versus public diabetes care in Iran from a payer perspective. This is the first study to evaluate these two models of diabetes care in Iran, to the best of our knowledge. The results of 20-year modelling showed that private diabetes care was associated with a % 3.2 higher LE life expectancy when compared to the public diabetes care (0.22 QALE gained). However, this higher LE was achieved by increasing the cost by %106. Our findings showed that %83 of diabetes costs in private setting were complication management costs. Other studies have shown that diabetes hospital care (mostly due to the diabetes complications) comprises the largest share of diabetes direct medical costs [28, 29]. Another study in 2011 found that the cost of inpatient services of T2DM in the private sector is 1.5 times higher than in the public sector [30]. As our results also confirm, this gap is expected to widen day over time [31]. These findings show that the occurrence and prevalence of complications and therefore their management costs can effectively affect the total costs of diabetes. Therefore, if the private sector can significantly increase the quality of its care and decrease the incidence of diabetes complications, we can expect to reduce the costs of complication management. This can lead to providing more cost-effective diabetes care.

T2DM is one of the Iran’s leading causes of morbidity and mortality and consumes nearly 8.7% of overall health spending [32, 33]. In recent years many private diabetes care developed in Iran to meet the needs of the patients with T2DM. Although there are no official statistics to show the percentage of the diabetes patients who are using private centers to receive their required medical care, our study showed that in larger provinces, like Tehran and Isfahan, more than %50 of the diabetes patients have registered in the private sector. Almost all of them expect to receive quality care compared to public diabetes care.

 The quality of medical services and patient outcomes influenced by various (internal and external) factors such as resource availability, patient engagement, and provider collaboration. The limited time available to each patient often leads to prescribing a blood test, and other human aspects of effective diabetes management (such as patient education, individual therapy, and diabetes self-management) are ignored [34]. It is stated that public diabetes clinics are often overcrowded, leading to prolonged waiting times and reduced face-to-face communication time between patient and physician [35]. However, our results showed that when considering the final outcome of diabetes care, QALE, the quality of public diabetes centers is quite acceptable. Another study in South Africa, also indicated that, contrary to expectations, Health Quality of life (HrQoL) and quality of care found to be similar across the two settings, especially about T2DM-related complications [13].

Our study has some limitations. Our sample size (1978 patients) is relatively small, compared to the total diabetic patients in Iran (more than 6 million patients) and therefore, the generalizability of the results should be cautious. The second point is that the UKPDS model does not explicitly include several diabetes-related morbidities (e.g., peripheral neuropathy); as a result, the use of the UKPDS model may result in the slightly overestimated ICER [36].

## Conclusion

Diabetes care in private centers is associated with a slight increase in the LE of T2DM patients. However, this higher LE was achieved by doubling the costs. Given the cost and and the outcomes of diabetes care, the quality of public diabetes centers is acceptable. The ICER indicated that the private diabetes cares are not cost-effective comparing to public diabetes care in the Iranian health care setting.

### Recommendations for future research directions

In order to increase the generalizability of results, Although methodologically challenging, it would be very useful to conduct some longer-term studies which sought to quantify the qualiy of cares in other provinces of Iran that maybe have different care patterns in diabetes.

## Data Availability

Extra data is available by email to the corresponding author on reasonable request.

## Footnotes

1 United Kingdom Prospective Diabetes Study

## References

[1] Esteghamati A, Etemad K, Koohpayehzadeh J, Abbasi M, Meysamie A, Noshad S (2014). Trends in the prevalence of diabetes and impaired fasting glucose in association with obesity in Iran: 2005-2011. Diabetes Res Clin Pract.

[2] Afarideh M, Ghajar A, Noshad S, Saadat M, Khajeh E, Esteghamati A (2017). Serum 25-hydroxyvitamin D, non-alcoholic fatty liver disease and type 2 diabetes. Nutr Metab Cardiovasc Dis.

[3] Zarei R, Anvari P, Eslami Y, Fakhraie G, Mohammadi M, Jamali A (2017). Retinal nerve fibre layer thickness is reduced in metabolic syndrome. Diabet Med.

[4] Vasheghani-Farahani A, Hosseini K, Ashraf H, Abolhasani M, Karbalai S, Ghajar A (2017). Correlation of ankle-brachial index and peripheral artery disease with the status of body fat deposition and metabolic syndrome in asymptomatic premenopausal women. Diabetes Metab Syndr.

[5] Association AD (2016). 8. Cardiovascular disease and risk management. Diabetes Care.

[6] Delavari A, Alikhani S, Nili S, Birjandi RH, Birjandi F. Quality of care of diabetes mellitus type II patients in Iran. Arch Iran Med. 2009;12(5): 492 – 495. 19722773

[7] Nicolucci A, Greenfield S, Mattke S (2006). Selecting indicators for the quality of diabetes care at the health systems level in OECD countries. Int J Qual Health Care.

[8] (ABIM) ABoIM (2015). Number of Candidates Certified.

[9] Gray PA, Drayton-Brooks S, Williamson KM (2013). Diabetes: follow-up support for patients with uncontrolled diabetes. Nurse Pract.

[10] Calsbeek H, Ketelaar NA, Faber MJ, Wensing M, Braspenning J (2013). Performance measurements in diabetes care: the complex task of selecting quality indicators. Int J Qual Health Care.

[11] White RO, Beech BM, Miller S (2009). Health care disparities and diabetes care: practical considerations for primary care providers. Clin Diabetes.

[12] Wabo NC, Örtenwall P, Khorram-Manesh A (2012). Hospital evacuation; planning, assessment, performance and evaluation. J Acute Disease.

[13] Pinchevsky Y, Raal F, Butkow N, Chirwa T, Distiller L, Rothberg A (2018). Quality of care delivered to type 2 diabetes mellitus patients in public and private sector facilities in Johannesburg, South Africa. Int J Gen Med.

[14] Baudot FO, Aguade AS, Barnay T, Gastaldi-Menager C, Fagot-Campagna A (2019). Impact of type 2 diabetes on health expenditure: estimation based on individual administrative data. Eur J Health Econ.

[15] Azam IS, Khuwaja AK, Rafique G, White F (2010). Assessment of quality of care for the management of type 2 diabetes: a multicentre study from a developing country. Qual Prim Care.

[16] Bhojani U, Devedasan N, Mishra A, De Henauw S, Kolsteren P, Criel B (2014). Health system challenges in organizing quality diabetes Care for Urban Poor in South India. Plos One.

[17] (MOHME) MoHaME. statistics and information of hospitals site in Iran. Available from: https://behdasht.gov.ir/rbp

[18] Vali L, Tabatabaee SS, Kalhor R, Amini S, Kiaei MZ (2016). Analysis of productivity improvement act for clinical staff working in the health system: a qualitative study. Global J Health Sci.

[19] Sadeghi A, Barati O, Bastani P, Jafari DD, Etemadian M (2016). Experiences of selected countries in the use of public-private partnership in hospital services provision. J Pak Med Assoc.

[20] Alavinia SMGM, Mahdavi Hezaveh A, Kermanchi J, Nasli Esfahani E, Yarahmadi S (2012). Tehran. National comprehensive program for prevention and control of diabetes mellitus Type II in urban areas.

[21] Ravaghi H, Sajadi HS, Ghotbi M, Sarvarizadeh S, Sharbafchizadeh N, Kermanchi J (2014). Evaluation of an urban phase of the specialized care program for diabetes in Iran: providers’ perspectives. Int J Prev Med.

[22] Mehrdad R (2009). Health system in Iran. JMAJ.

[23] Hayes AJ, Leal J, Gray A, Holman R, Clarke P (2013). UKPDS outcomes model 2: a new version of a model to simulate lifetime health outcomes of patients with type 2 diabetes mellitus using data from the 30 year United Kingdom prospective diabetes study: UKPDS 82. Diabetologia.

[24] Brown DS, Jia H, Zack MM, Thompson WW, Haddix AC, Kaplan RM (2013). Using health-related quality of life and quality-adjusted life expectancy for effective public health surveillance and prevention. Expert Rev Pharmacoecon Outcomes Res.

[25] Jia H, Zack MM, Thompson WW (2011). State Quality-Adjusted Life Expectancy for US adults from 1993 to 2008. Qual Life Res.

[26] Brooks R (1996). EuroQol: the current state of play. Health Policy (Amsterdam, Netherlands).

[27] Group TE. EuroQol-a new facility for the measurement of health-related quality of life. Health policy. 1990;16(3):199-208. 10.1016/0168-8510(90)90421-9 10109801

[28] Javanbakht M, Mashayekhi A, Baradaran HR, Haghdoost A, Afshin A (2015). Projection of diabetes population size and associated economic burden through 2030 in Iran: evidence from Micro-simulation Markov model and Bayesian Meta-analysis. Plos One.

[29] Javanbakht M, Baradaran HR, Mashayekhi A, Haghdoost AA, Khamseh ME, Kharazmi E (2011). Cost-of-illness analysis of type 2 diabetes mellitus in Iran. Plos One.

[30] Esteghamati A, Khalilzadeh O, Anvari M, Meysamie A, Abbasi M, Forouzanfar M (2009). The economic costs of diabetes: a population-based study in Tehran, Iran. Diabetologia.

[31] Liebl A, Khunti K, Orozco-Beltran D, Yale J-F (2015). Health economic evaluation of type 2 diabetes mellitus: a clinical practice focused review. Clin Med Insights Endocrinol Diabetes.

[32] Davari M, Boroumand Z, Amini M, Aslani A, Hosseini M (2016). The direct medical costs of outpatient cares of type 2 diabetes in Iran: a retrospective study. Int J Prev Med.

[33] Doshmangir L, Rashidian A, Ravaghi H, Takian A, Jafari M. The experience of implementing the board of trustees’ policy in teaching hospitals in Iran: an example of health system decentralization. International Journal of Health Policy and Management. 2015;4(4):207. 10.15171/ijhpm.2014.115 PMC4380562 25844379

[34] Aeenparast A, Farzadi F, Maftoon F (2012). Waiting time for specialist consultation in Tehran. Arch Iran Med.

[35] Noshad S, Afarideh M, Heidari B, Mechanick JI, Esteghamati A (2015). Diabetes care in Iran: where we stand and where we are headed. Ann Glob Health.

[36] Clarke PM, Gray AM, Briggs A, Farmer AJ, Fenn P, Stevens RJ (2004). A model to estimate the lifetime health outcomes of patients with type 2 diabetes: the United Kingdom prospective diabetes study (UKPDS) outcomes model (UKPDS no. 68). Diabetologia.

